# Maintenance of muscle strength retains a normal metabolic cost in simulated walking after transtibial limb loss

**DOI:** 10.1371/journal.pone.0191310

**Published:** 2018-01-12

**Authors:** Elizabeth Russell Esposito, Ross H. Miller

**Affiliations:** 1 Center for the Intrepid, Brooke Army Medical Center, Department of Rehabilitation Medicine, JBSA, Ft. Sam Houston, Texas, United States of America; 2 Extremity Trauma and Amputation Center of Excellence, Ft. Sam Houston, Texas, United States of America; 3 Department of Kinesiology, University of Maryland, College Park, Maryland, United States of America; 4 Neuroscience & Cognitive Science Program, University of Maryland, College Park, Maryland, United States of America; Northwestern University, UNITED STATES

## Abstract

Recent studies on relatively young and fit individuals with limb loss suggest that maintaining muscle strength after limb loss may mitigate the high metabolic cost of walking typically seen in the larger general limb loss population. However, these data are cross-sectional and the muscle strength prior to limb loss is unknown, and it is therefore difficult to draw causal inferences on changes in strength and gait energetics. Here we used musculoskeletal modeling and optimal control simulations to perform a longitudinal study (25 virtual “subjects”) of the metabolic cost of walking pre- and post-limb loss (unilateral transtibial). Simulations of walking were first performed pre-limb loss on a model with two intact biological legs, then post-limb loss on a model with a unilateral transtibial prosthesis, with a cost function that minimized the weighted sum of gait deviations plus metabolic cost. Metabolic costs were compared pre- vs. post-limb loss, with systematic modifications to the muscle strength and prosthesis type (passive, powered) in the post-limb loss model. The metabolic cost prior to limb loss was 3.44±0.13 J/m/kg. After limb loss, with a passive prosthesis the metabolic cost did not increase above the pre-limb loss cost if pre-limb loss muscle strength was maintained (mean -0.6%, *p* = 0.17, *d* = 0.17). With 10% strength loss the metabolic cost with the passive prosthesis increased (mean +5.9%, *p* < 0.001, *d* = 1.61). With a powered prosthesis, the metabolic cost was at or below the pre-limb loss cost for all subjects with strength losses of 10% and 20%, but increased for all subjects with strength loss of 30% (mean +5.9%, *p* < 0.001, *d* = 1.59). The results suggest that maintaining muscle strength may prevent an increase in the metabolic cost of walking following unilateral transtibial limb loss, and that a gait with minimal deviations can be achieved when muscle strength is sufficiently high, even when using a passive prosthesis.

## Introduction

Individuals with lower limb loss face a variety of issues that can limit their mobility, affecting quality of life and risk for various secondary conditions [1]. One such issue is the metabolic cost of walking, which is often greater in individuals with limb loss than in those without [2]. In individuals with unilateral transtibial limb loss, different passive prosthesis designs or stiffness settings have provided modest metabolic cost reductions at best [3,4] and results to date from powered prostheses have been mixed [5–7]. These results are surprising, given that optimal control simulations have suggested prosthesis stiffness and power can theoretically reduce the metabolic cost of walking by greater amounts [8,9].

When prosthesis design alone does not appear to fully account for the metabolic cost of walking with limb loss, another potential contributing factor that has received less attention is fitness (e.g. muscle strength). When subjects with and without limb loss are closely matched for age and fitness, they often walk with similar metabolic costs. For example, relatively young military Service Members with limb loss due to traumatic injuries can walk with a passive transtibial prosthesis with metabolic costs that do not differ significantly from able-bodied Service Members [10,11]. “Young” in the limb loss population typically refers to ages ~ 18–44 years [12], or well below the typical “Older Adult” threshold of 60–65 years. Subject age in [10] and [11] averaged 28 years with range 23–41 years. A potential mechanism for these results is that while limb loss removes the ability of the lost muscles to directly perform mechanical work at the ankle, which may necessitate energetically costly compensations elsewhere, it also removes the ability of these muscles to consume metabolic energy, and the ankle plantarflexors account for about a quarter of the total metabolic cost of walking [13]. With sufficient fitness, perhaps these energy savings outweigh the costs of compensatory adjustments.

This mechanism is difficult to test in human subjects due to the obvious difficulties in obtaining longitudinal data pre- and post-limb loss. Consequently, the effect of maintaining pre-limb loss muscle strength on the metabolic cost of walking post-limb loss is unknown. Optimal control simulations can be useful in such situations where obtaining data from live humans is impractical or impossible [14] and in situations where multiple objectives are relevant in the control problem, e.g. metabolic cost, gait deviations, symmetry, balance, joint loading, etc. [15]. Several previous studies have used optimal control methods [8,9,15–18] and other related methods [19] to simulate limb loss gait, but have not modeled changes in muscle strength with limb loss, and it is unknown how various levels of bilateral or unilateral (residual limb) muscle strength loss affect the metabolic cost of walking post-limb loss.

Therefore, the purpose of this study was to determine the effect of muscle strength on the metabolic cost of optimal control simulations of walking, following unilateral transtibial limb loss. Simulations were performed emulating the common limb loss rehabilitation and prosthesis-fitting goal of minimizing deviations from typical non-amputee gait mechanics [20,21]. Based on recent evidence from the military Service Member population [10,11], we hypothesized that limb loss and use of a passive transtibial prosthesis would only increase the mass-specific metabolic cost of walking (energy per unit distance, per unit biological body mass) if the muscle strength of the rest of the body that remains after limb loss was reduced. As secondary analyses, we examined how prosthesis design (passive stiffness, and passive vs. powered actuation) affects the consequences of strength loss on the metabolic cost of walking with a transtibial prosthesis.

## Methods

### Pre-limb loss model description

**Fig 1:**
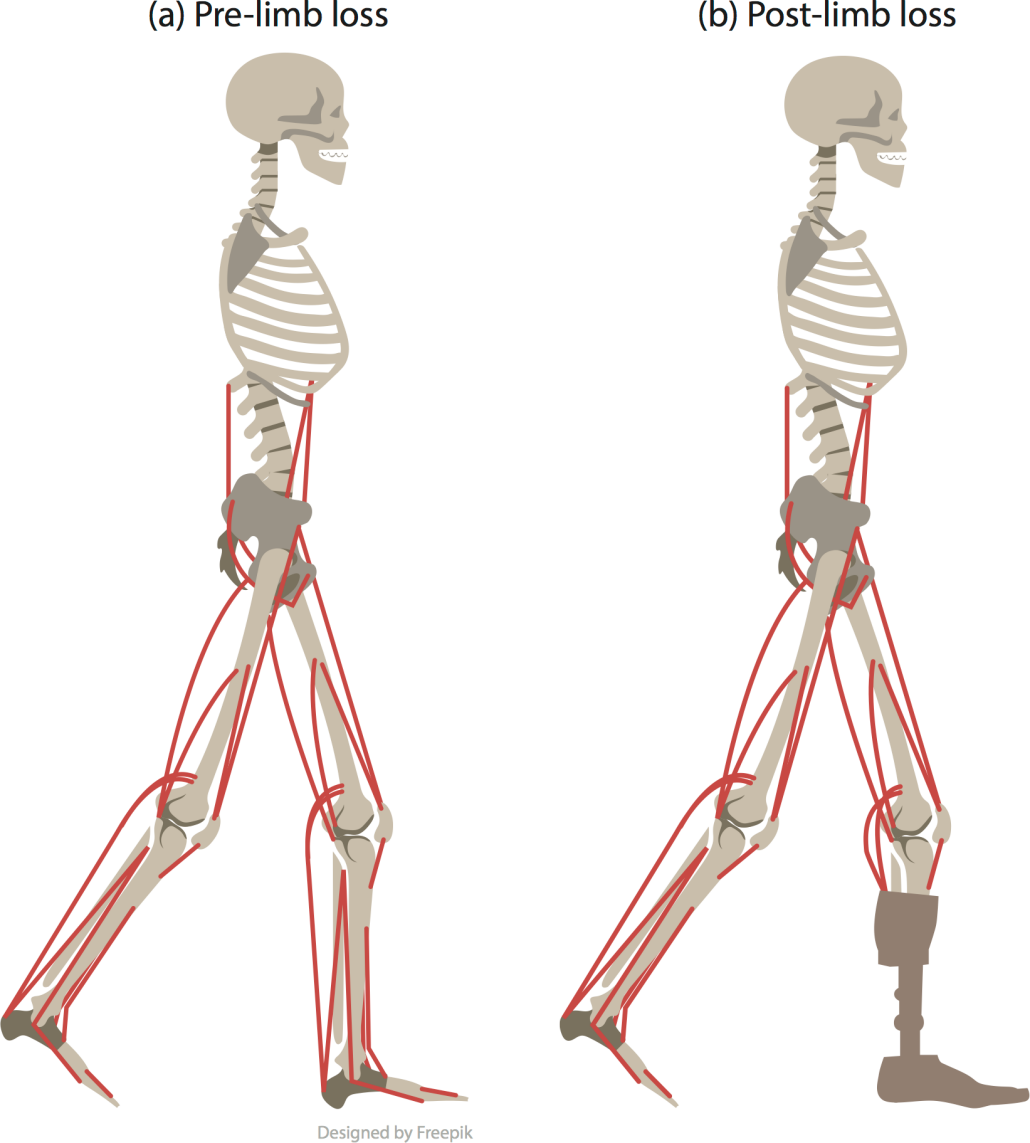
Model drawings. Conceptual drawings of (a) the pre-limb loss model and (b) the post-limb loss model, with the right ankle muscles removed and replaced with a transtibial prosthesis.

Simulations of walking were performed using a 2D sagittal plane computer model (Fig 1). The model was based on an earlier model used to simulate running [22] and was conceptually similar to other models used for these types of simulations [8,9,15,16]. Full technical details on the present model’s formulation are presented in Supplemental Information (S3 File). Briefly, the model consisted of 10 rigid segments (pelvis, trunk, thighs, shanks, feet, and toes) connected at nine joints. The joints were actuated by 26 Hill-based muscle models representing the major flexors/extensors of the trunk and lower limbs: erector spinae, rectus abdominus, iliopsoas, glutei, rectus femoris, hamstrings, vasti, biceps femoris (short head), gastrocnemius, tibialis anterior, soleus, extensor digitorum, and flexor digitorum. Muscle model parameters were defined to represent the lower limb joint strength of a healthy young adult male [23]. Muscle metabolic rates were calculated as a function of contractile component activation and velocity [24].

### Post-limb loss model description

**Fig 2:**
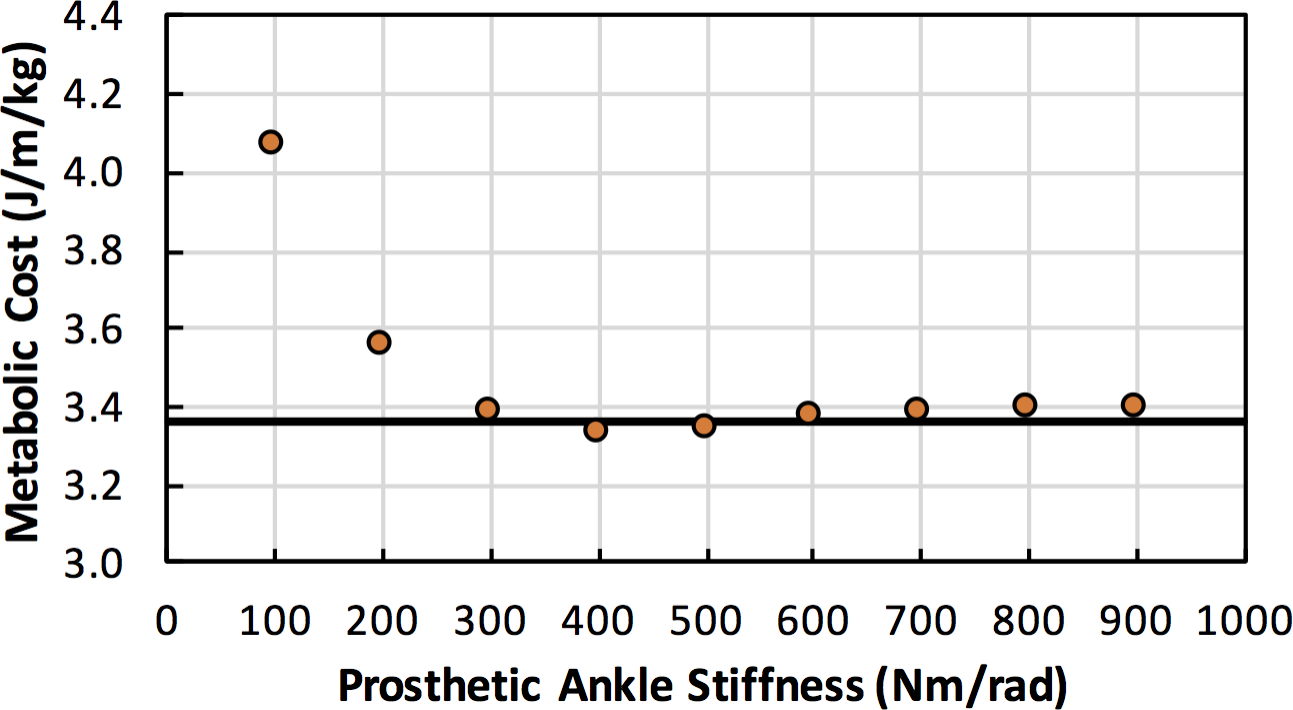
Prosthetic ankle stiffness vs. metabolic cost. Metabolic cost of walking at 1.3 m/s using the post-limb loss model with no strength loss and a passive prosthesis, with different prosthetic ankle stiffnesses. Horizontal line is the metabolic cost of the pre-limb loss simulation at this same speed.

In the post-limb loss model, the five ankle muscles of the right leg (gastrocnemius, tibialis anterior, soleus, extensor digitorum, and flexor digitorum) were removed, as other similar models have done [8,9,15,16], and the right ankle’s passive torque-angle relationship [25] was replaced with a linear relationship representing a passive prosthesis [15,16]:

τ=−k∙q (1)

where *τ* and *q* are the respective torque and angle of the right ankle. The angle *q* = 0 corresponded to the ankle angle during upright standing, with the foot flat on the ground and the shank perpendicular to the ground. The torsional stiffness *k* = 400 Nm/rad was selected from sensitivity analyses as the value that produced the lowest metabolic cost in walking simulations (Fig 2), and is within the range of stiffnesses for typical commercially-available dynamic response prostheses [26]. The mass and moment of inertia of the prosthesis were reduced to 65% and 40%, respectively, of the biological limb values to represent a typical transtibial prosthesis [27]. To avoid underestimating the mass-specific metabolic cost in the limb Loss model, the entirety of the shank segment’s mass on the residual limb was conservatively assumed to be non-biological. The terms in the right knee’s passive torque-angle relationship that depended on the ankle angle were also removed. The pre- and post-limb loss models were otherwise identical.

This approach to modeling transtibial limb loss neglects the fact that ankle muscles may be partially retained in transtibial amputation surgery, depending on the level of amputation, to pad the end of the residual limb, but it is unclear how the surgery affects the mechanics and control of these muscles, making them difficult to model with confidence. The post-limb loss model could still actively control flexion of the residual limb’s knee through biceps femoris (short head), and the same statistically significant changes in metabolic cost pre- vs. post-limb loss reported here were seen when gastrocnemius was retained as a uniarticular knee flexor. These results were omitted here for brevity and focus.

### Simulations

#### Subjects

Since this study was a computer simulation study, the “subjects” were instances of the model described in §2.1–2.2 with different sets of parameter values. The models were not intended to represent any specific individuals, but rather a hypothetical population of healthy young adult males. Uniform distributions for muscle moment arm lengths, maximum isometric forces, fast-twitch fiber fractions, and unloaded series elastic component lengths were defined centered on the original value with a width of ±10%, and new sets of parameter values were drawn randomly from these distributions to define a sample of 25 subjects. These particular parameters were used to define subjects because model results can be particularly sensitive to their values [14,28,29]. The 10% distribution width is a reasonable estimate of the variance between human subjects in these parameters [30].

The sample size was chosen based on pilot data from a preliminary sample of 20 simulated subjects that suggested a sample size of 25 could detect differences in metabolic cost with and without limb loss of 9–33% as statistically significant (*p* < 0.05) with 80–99% power. This range (9–33%) is the range typically reported by studies on the general population of individuals with unilateral transtibial limb loss [10]. The study was therefore powered to detect differences of the size seen in the general population as “significant” and differences of the size seen in the Service Member population as “insignificant”, similar to results from gait experiments with human subjects. The approach to modeling limb loss (§2.2) was the same for all subjects.

#### Overview

A total of 385 simulations were performed: 10 validation simulations, plus 25 subjects with 15 simulations per subject. In all simulations, the models described in §2.1–2.2 were used to simulate a periodic stride of walking with stride duration *T* and average speed *v*. The simulations were cast as optimal control problems: for a model **f** with muscle excitations **u**(*t*), state variables **x**(*t*), and prescribed speed *v*, determine the values of **u**(*t*), **x**(*t*), and *T* that minimize a user-defined cost function *J*. The cost function for all simulations was the weighted sum of a tracking error (first term) plus the metabolic cost (second term):

$$J=\frac{1}{13T}\sum_{i=1}^{13}\int_{0}^{T}{\left(\frac{x_{i}(t)-\mu_{i}(t)}{\sigma_{i}(t)}\right)}^{2}dt+w{\left(\frac{1}{mvT}\sum_{i=1}^{26}\int_{0}^{T}{\dot{E}}_{i}(t)dt\right)}^{2}$$ (2)

In the tracking term, *x*_*i*_(*t*) is the value of model variable *i* at time *t*, *μ*_*i*_(*t*) is the between-subjects mean of this same variable from human experimental gait data, and *σ*_*i*_(*t*) is the between-subjects standard deviation (SD) of *μ*_*i*_(*t*) [31]. In the metabolic cost term, *m* is the biological body mass, ${\dot{E}}_{i}(t)$ is the metabolic rate of muscle *i* at time *t*, and *w* is a weighting constant. The 13 specific variables included in the tracking term were the pelvis angle, the angles of the back, hip, knee, and ankle joints, the vertical and horizontal ground reaction forces (GRF), and the stride duration. The square root of the first term in Eq 2 gives the average tracking error, in multiples of SD. For example, a tracking error of 1.5 indicates the model’s gait deviations were 1.5 SD away from the experimental means when averaged over all timesteps of all tracking targets. A value of *w* = 0.1 was found to produce realistic metabolic costs and timing of muscle activity, with tracking errors typically under two SD at all timesteps, and was used in all simulations.

The optimal control problems were converted to nonlinear programming problems on a temporal grid of 101 nodes per stride, and solved using direct collocation [22,32]. Technical details are included in the Supplemental Information (S3 File).

#### Validation simulations

Modeling and simulation are arguably most useful for their ability to predict results from conditions that are impractical or impossible to study with experiments on real humans. For example, here we studied the impractical condition of measuring the metabolic cost of walking before and after limb loss. To gain confidence in the model’s ability to make such predictions, it is helpful to test its accuracy when predicting an expected result. To this end, we tested the ability of the pre- and post-limb loss models with their original parameter values to predict the relationship between walking speed and metabolic cost. Simulations at five speeds between *v* ∈ [0.73,1.67] m/s were performed using means and standard deviations of experimental kinematics and GRF from young Service Members with and without unilateral transtibial limb loss for *μ*_*i*_(*t*) and *σ*_*i*_(*t*) in the cost function [10].

**Fig 3:**
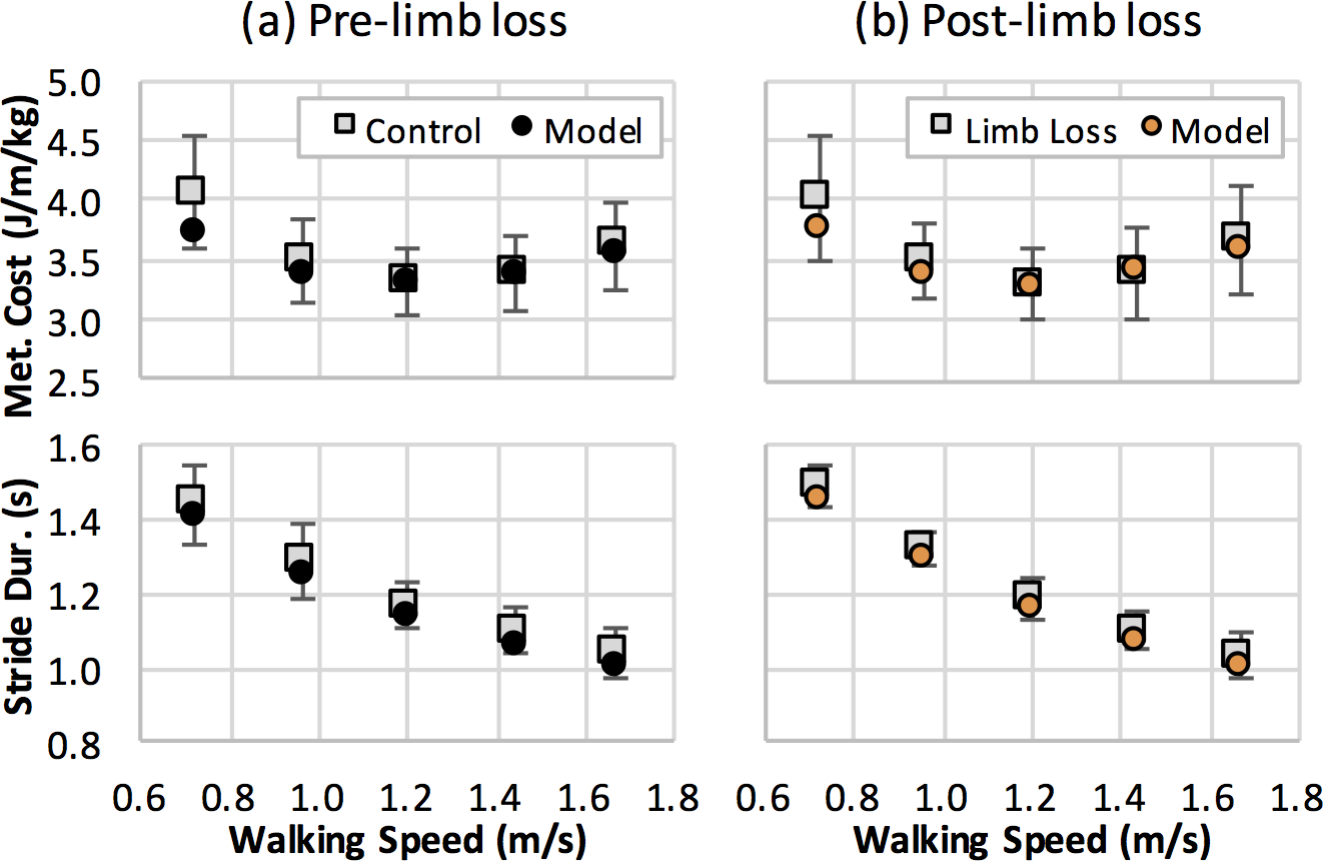
Model validity. Metabolic cost and stride duration for simulations of walking at five different speeds (circles) using (a) the pre-limb loss model and (b) the post-limb loss model. The squares and error bars are means and standard deviations for human experimental data [10] with (a) Service Members with two intact limbs and (b) Service Members with unilateral transtibial limb loss walking with a passive transtibial prosthesis.

Both the pre- and post-limb loss models produced the expected *U*-shaped relationship between metabolic cost and walking speed, and the metabolic costs and stride durations fell within one standard deviation of the experimental means at all speeds for both models (Fig 3). Note that the models were not attempting to track the experimental metabolic cost data in Fig 3: the model-based metabolic costs were produced by minimizing the cost function (Eq 2) at each prescribed speed, without the optimizer knowing what the experimental metabolic costs were.

#### Pre-limb loss walking simulations

For each subject, the pre-limb loss version of the model (§2.1) was used to simulate a periodic stride of walking with average speed *v* = 1.3 m/s. The between-subjects means and standard deviations of experimental kinematics and GRF from 14 healthy adults without limb loss walking in a “normal and comfortable” fashion [33] were used as the respective tracking targets *μ*_*i*_(*t*) and scaling factors *σ*_*i*_(*t*) in the cost function (Eq 2). The same tracking targets were used for all subjects, but the resulting gaits and metabolic costs differed between subjects since each subject had different model parameter values (§2.3.1).

#### Post-limb loss walking simulations

For each subject, the post-limb loss version of the model was used to perform a second simulation of walking, also at *v* = 1.3 m/s. The cost function was the same as the pre-limb loss simulations (Eq 2), with the same means and SDs from [33] in the tracking term. Thus, the goal of the post-limb loss simulations could be described as attempting to walk with minimal gait deviations, a common goal of rehabilitation and prosthesis fitting, with minimal metabolic cost. A “gait deviation” here is a substantial departure from the range of typical gait mechanics of individuals without limb loss. The weighting between the tracking term and the metabolic cost term (*w* = 0.1) was the same as the pre-limb loss simulations.

#### Hypothesis testing and statistics

To test the hypothesis that use of a passive transtibial prosthesis would not increase the metabolic cost of walking if the pre-limb loss muscle strength was maintained, the post-limb loss simulation was performed twice for each subject, once with the same maximum isometric muscle forces (*F*_*o*_) as the subject’s pre-limb loss model, and once with *F*_*o*_ reduced by 10% for all muscles, including the trunk muscles. This strength loss is relevant to simulate because individuals with limb loss often have muscle atrophy in both limbs, with losses of up to 25% [34], and it is also within the typical range of strength differences seen in young vs. older adults [30,35]. However, other magnitudes and types of strength loss (i.e. unilateral) were also simulated (see below).

Simulations with the weaker post-limb loss models were followed up by six additional simulations per subject to determine the effect of prosthesis design on the weaker model’s metabolic cost. In the first follow-up simulation, the simulations with 10% strength loss were repeated with the optimizer allowed to adjust the prosthetic ankle stiffness *k* (Eq 1) over the range of 100–800 Nm/rad to minimize the cost function, representing a prosthesis with subject-specific stiffness setting as could be accomplished in reality by prosthesis selection [26], adjustment of spring positions [4], or additive manufacturing [36]. In the other follow-up simulations, an actively-controlled term was added to the prosthesis torque-angle relationship to represent a powered prosthesis that could deliver time-varying plantarflexion torque:

$$\tau =-k∙q-a∙\tau_{max}$$ (3)

$$\dot{a}(t)=(i-a)(c_{1}i+c_{2})$$ (4)

where 0 ≤ *i*(*t*) ≤ 1 is a control variable analogous to a motor current, *a*(*t*) is the motor activation, *τ*_*max*_ = 500 Nm is the maximum torque, and *k* = 400 Nm/rad. The motor activation rate constants *c*_1_ = 5 s^-1^ and *c*_2_ = 30 s^-1^ produced smooth motor torque while allowing very fast activation dynamics, analogous to a 100% fast-twitch muscle [14,37]. The maximum torque was set simply to allow the prosthesis to produce a realistic range of torques for walking. The powered prosthesis control and activation signals did not appear in the cost function, so the value of *τ*_*max*_ does not affect the simulation results unless it is unrealistically low for gait.

Simulations with the powered prosthesis were performed with *F*_*o*_ reduced by 0%, 10%, 20%, and 30% vs. the pre-limb loss model. In these simulations, the optimizer could adjust the control *i*(*t*) of the powered prosthesis, along with the muscle excitations, to minimize the cost function. The simulations with 30% strength loss were also repeated with the optimizer again allowed to adjust the passive stiffness *k*. Each of the simulations involving strength loss was performed once with strength loss for all muscles in the model (bilateral strength loss), and once with strength loss for only the leg and trunk muscles on the side of the residual limb (unilateral strength loss). These conditions were tested because individuals with unilateral limb loss often have strength asymmetries in the range of 10–30%, with weaker muscles in the residual limb, especially if the individual is sedentary [38,39].

#### Statistical analysis

The outcome variable for each simulation was the gross metabolic cost, expressed as J/m per kg biological mass and calculated assuming a basal metabolic rate of 1.0 W per kg biological mass. Differences in metabolic cost between the pre- and post-limb loss simulations were assessed by two-tailed matched-pair Student’s *t*-tests (critical *p* = 0.05/14 after Bonferonni correction) and were complemented by Cohen’s *d* effect sizes and Bayes factors (*B*). Effect sizes were calculated using the between-subjects standard deviation of the pre-limb loss simulations in the denominator. Effect size *d* > 0.80 is typically deemed a “large” effect [40]. Bayes factors were the ratio of likelihood probabilities for the alternative hypothesis (difference in metabolic costs) vs. the null hypothesis (no difference), and were calculated using the JZS prior with scale *r* = 1.0 [41]. Bayes factor *B* > 100 is typically deemed “decisive” evidence in favor of the alternative hypothesis, while *B* < 3 is “barely worth mentioning” [42].

## Results

### Gait mechanics

**Fig 4:**
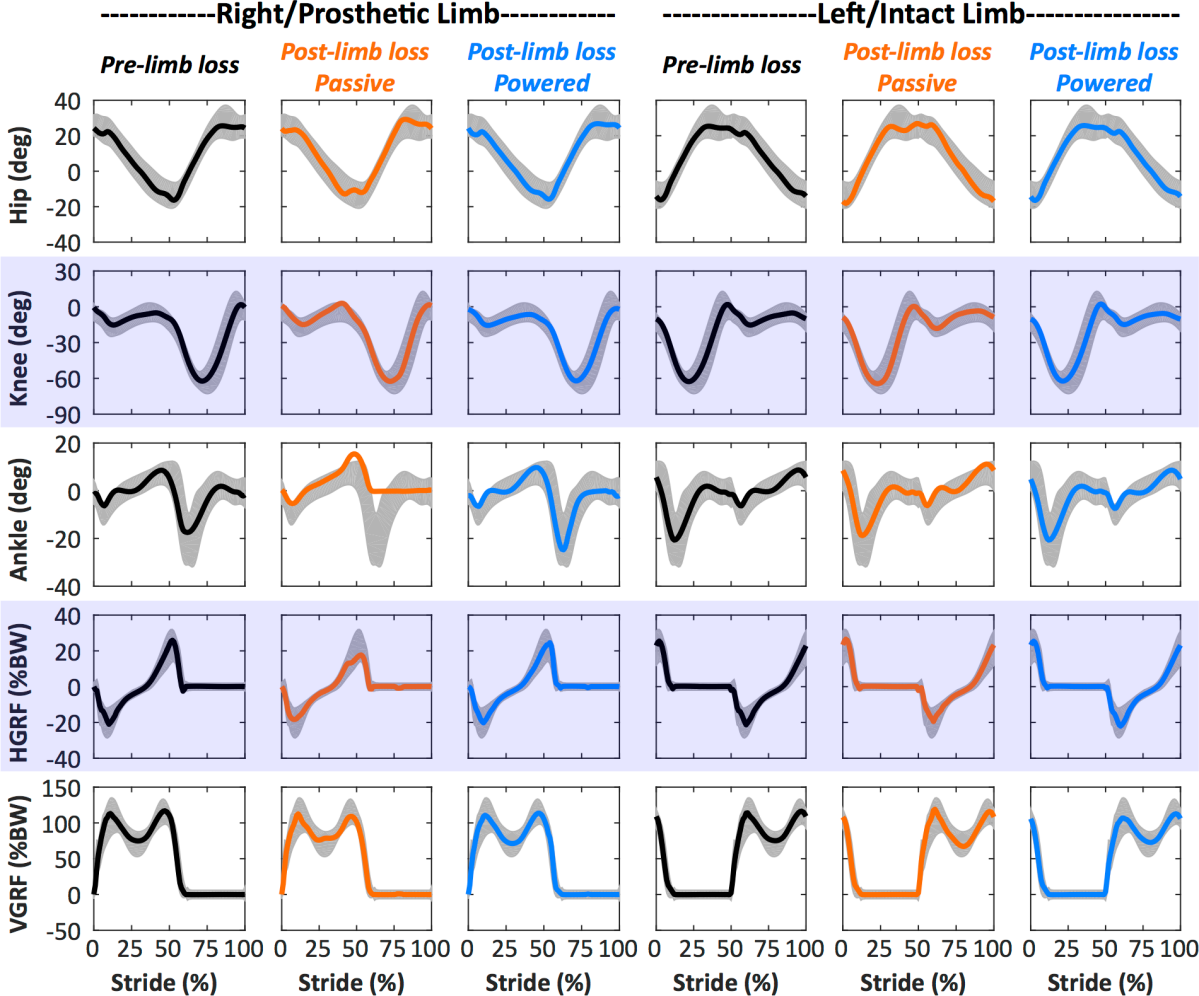
Example gait mechanics. Gait kinematics (hip, knee, and ankle angles) and kinetics (horizontal and vertical ground reaction forces, HGRF and VGRF) of Subject 01’s walking simulations with the pre-limb loss model (columns 1 and 4), and with the post-limb loss model using the passive prosthesis (columns 2 and 5) and the powered prosthesis (columns 3 and 6). The post-limb loss model had no strength loss. The stride begins at heel-strike of the right/residual limb. The shaded area in each panel is ± two standard deviations around the mean of experimental data for *N* = 14 healthy adults without limb loss [33].

**Fig 5:**
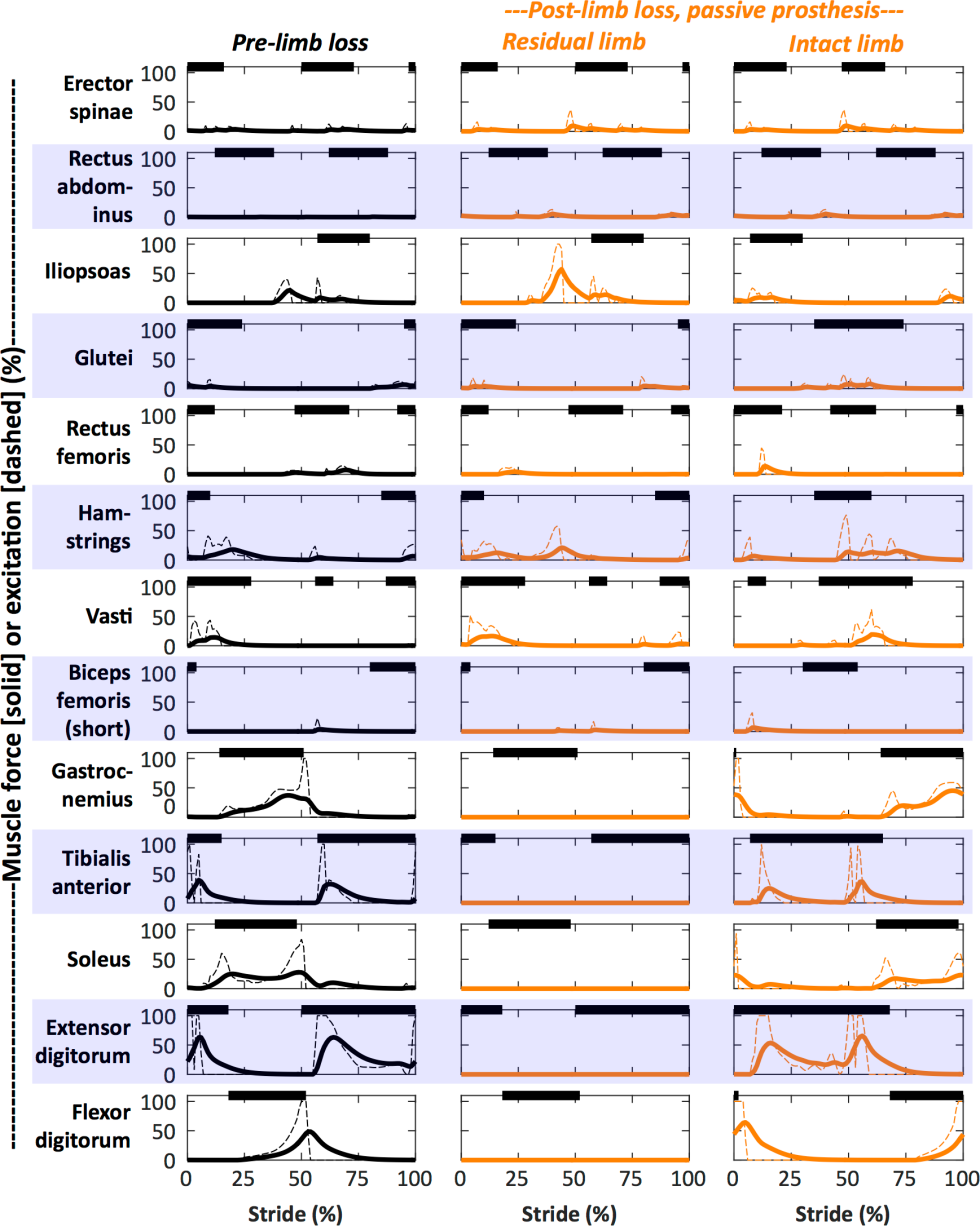
Example muscle forces and excitations. Muscle forces (solid lines, % max isometric force) and excitations (dashed lines) of Subject 01’s walking simulations with the pre-limb loss model (column 1) and with the post-limb loss model using the passive prosthesis (columns 2 and 3). The post-limb loss model had no strength loss. The stride begins at heel-strike of the right/residual limb. The thick bars at the top of each panel are typical “on/off” timing derived from experimental electromyograms [43,44].

Figs 4 and 5 show typical (Subject 01) gait mechanics (Fig 4) and muscle forces and excitations (Fig 5) for pre- and post-limb loss simulations with no strength loss. Animations of the mechanics data are included in the Supplemental Information (S1 and S2 Files). The simulated joint angles and GRF were typically within two SD of means from the referenced experimental gait data [33], with the expected exception of the passive prosthetic ankle angle. The timing of muscle excitations in the pre-limb loss simulations was similar in most cases to referenced normative electromyograms [43,44]. The differences in muscle excitations between the pre- and post-limb loss simulations were similar to the most consistently reported differences between amputees and non-amputees, including increased residual limb hip flexor activity and prolonged residual limb hamstrings activity [17].

**Fig 6:**
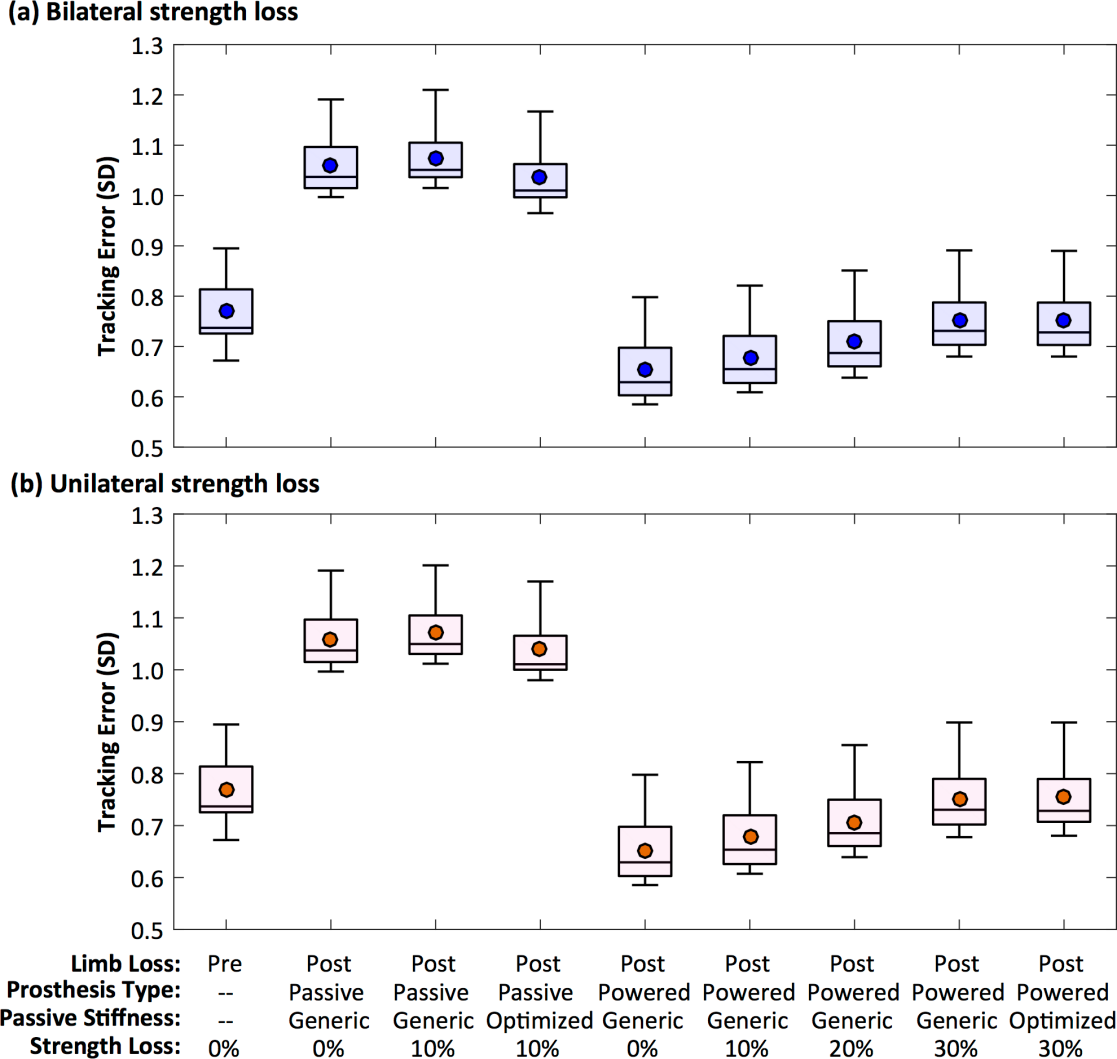
Tracking errors. Box plots of tracking errors for different prosthesis types, passive prosthetic ankle stiffness, and level of strength loss. Panel (a) is simulations of bilateral strength loss. Panel (b) is simulations of unilateral strength loss, on the side of the residual limb. The symbol within each box is the mean.

Fig 6 shows the average tracking errors / gait deviations for the pre- and post-limb loss simulations. Deviations were expectedly smaller when using the powered prosthesis than when using the passive prosthesis, due primarily to the limited range of motion of the passive prosthesis and its inability to perform net positive joint work. Optimization of the prosthetic ankle stiffness had a small but consistent effect of reducing gait deviations with the passive prosthesis, but did not affect gait deviations with the powered prosthesis. Strength loss systematically increased gait deviations, but the effect of strength loss on gait deviations was similar regardless of whether the strength loss was unilateral or bilateral.

### Metabolic costs of limb loss and prosthesis use

**Fig 7:**
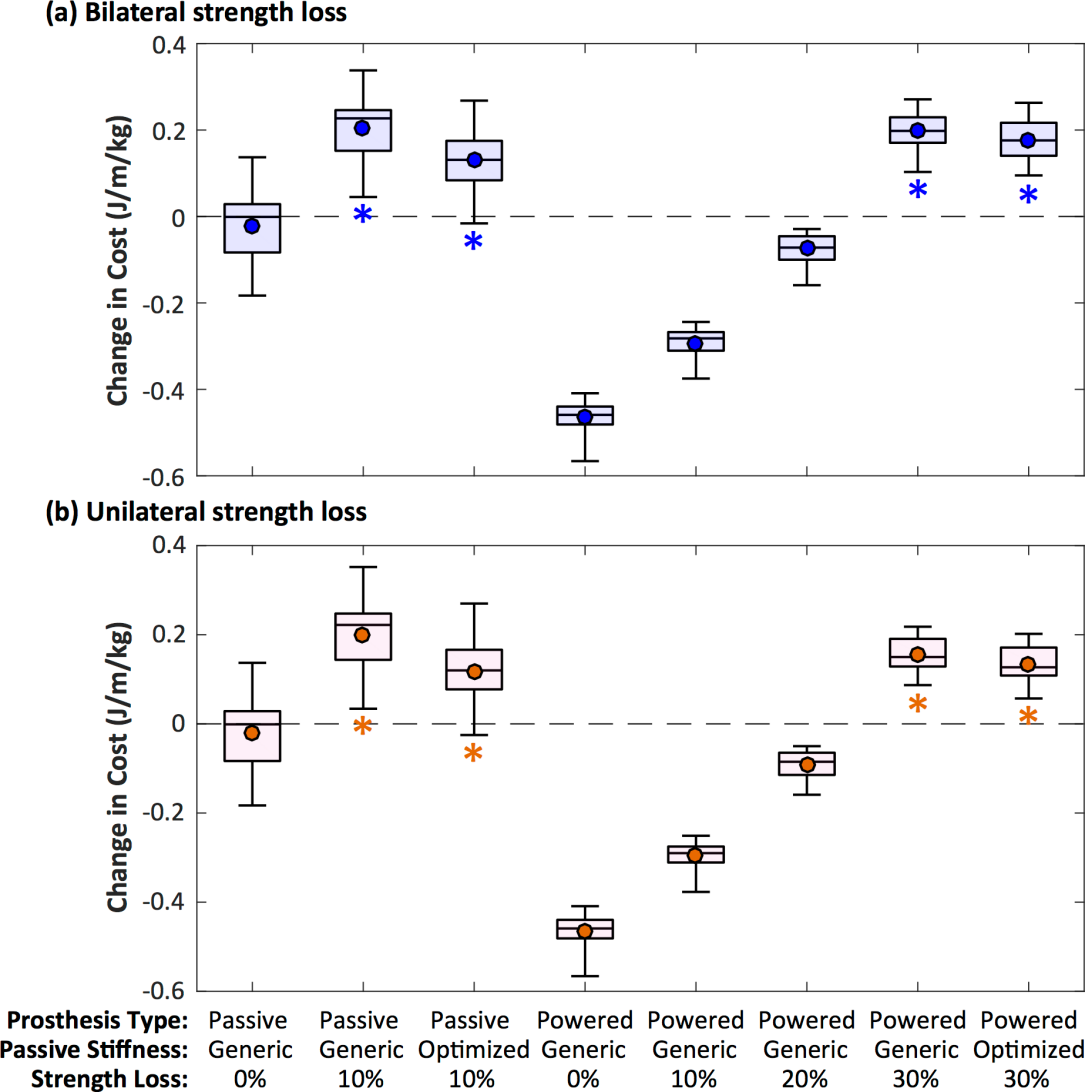
Changes in gross metabolic cost. Box plots of changes in metabolic cost of walking at 1.3 m/s (relative to the pre-limb loss simulation) for different prosthesis types, passive prosthetic ankle stiffness, and level of strength loss. Panel (a) is simulations of bilateral strength loss. Panel (b) is simulations of unilateral strength loss, on the side of the residual limb. The symbol within each box is the mean. * indicates the change was significantly greater than zero (*p* < 0.001, *d* > 1.0, *B* > 100).

The metabolic cost of the pre-limb loss models averaged 3.44±0.13 J/m/kg (range 3.20–3.68 J/m/kg), which compares well to typical human metabolic costs of 3.0–3.7 J/m/kg when walking near the speed of 1.30 m/s [45]. Changes in metabolic costs post-limb loss are presented in Fig 7.

### Passive prosthesis

With no strength loss, metabolic cost did not increase post-limb loss when using the passive prosthesis (mean±95%CI: -0.02±0.03 J/m/kg below pre-limb loss, *p* = 0.17, *d* = 0.17, *B* = 0.39). With 10% bilateral muscle strength loss post-limb loss, metabolic cost increased when using both generic passive prosthesis stiffness (+0.20±0.03 J/m/kg above pre-limb loss; *p* < 0.001, *d* = 1.61, *B* > 100) and optimized passive prosthesis stiffness (+0.13±0.03 J/m/kg above pre-limb loss; *p* < 0.001, *d* = 1.04, *B* > 100). Only one subject had a metabolic cost slightly below the pre-limb loss cost (-0.02 J/m/kg) when using the optimized passive prosthesis stiffness and 10% strength loss. Similar results were seen for 10% unilateral muscle strength loss (Fig 7).

**Fig 8:**
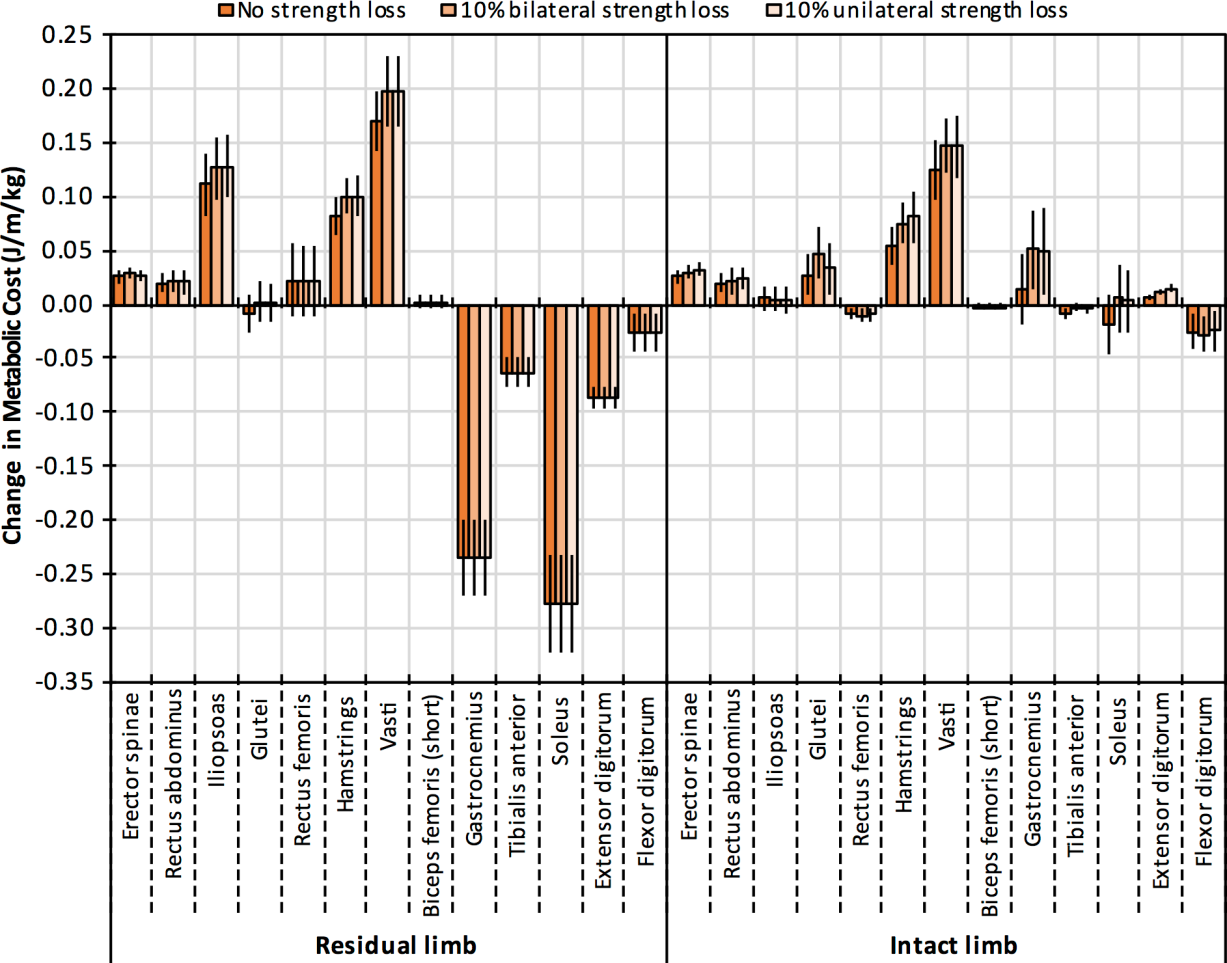
Changes in muscle metabolic costs. Changes in the metabolic cost of individual muscles in the model (mean±SD) between the pre- and post-limb loss simulations when using the passive prosthesis with generic stiffness and 10% strength loss.

Fig 8 shows average increases in the metabolic cost of individual muscles between the pre- and post-limb loss simulations when using the passive prosthesis. With 10% strength loss, the largest contributors to the increase in metabolic cost were iliopsoas, hamstrings, and vasti of the residual limb, and hamstrings, vasti, gastrocnemius, and soleus of the intact limb. Changes in muscle costs were similar between unilateral and bilateral strength loss, with the exception of glutei of the intact limb, which had a greater increase in cost with bilateral strength loss.

### Powered prosthesis

With no muscle strength loss, metabolic cost was reduced post-limb when using the powered prosthesis (-0.47±0.01 J/m/kg below pre-limb loss; *p* < 0.001, *d* = 3.71, *B* > 100). With 10% and 20% bilateral muscle strength loss, the metabolic cost post-limb loss was still below the pre-limb loss cost for all subjects when using the powered prosthesis. However, with 30% bilateral muscle strength loss, metabolic cost increased when using the powered prosthesis, both with generic passive stiffness (+0.20±0.02 J/m/kg above pre-limb loss; *p* < 0.001, *d* = 1.58, *B* > 100) and with optimized passive prosthesis stiffness (+0.18±0.02 J/m/kg above pre-limb loss; *p* < 0.001, *d* = 1.42, *B* > 100). Similar results were seen for unilateral muscle strength loss (Fig 7).

## Discussion

The purpose of the present study was to determine the effect of muscle strength loss following unilateral transtibial limb loss on the metabolic cost of walking. Several previous studies have used similar methods to simulate various practical and hypothetical aspects of amputee locomotion [8,9,15,16], but the present study is the first to our knowledge to model the muscle strength losses typically seen in the general limb loss population, as well as the maintenance of strength often seen in high-functioning individuals within this population [39,46]. In support of the hypothesis, the main result was that metabolic cost of optimal control simulations of walking with a passive transtibial prosthesis did not increase post-limb loss if pre-limb loss muscle strength was maintained. However, 10% strength loss significantly increased metabolic cost, even when only the residual limb had strength loss (Fig 7).

The mechanism for these results was straightforward and consistent with the hypothesis: muscles necessarily consume metabolic energy to generate force and perform work, and while transtibial amputation removes the ability of the lost ankle muscles to perform the net positive joint work seen at the ankle in unimpaired walking [47], it also removes their ability to consume metabolic energy. The five ankle muscles removed to model limb loss accounted for 20% of the gross metabolic cost on average in the pre-limb loss simulations. With no loss of muscle strength, the remaining muscles in the post-limb loss model collectively compensated for the lost muscles without increasing the gross metabolic cost. With 10% loss of muscle strength, more muscle activation was needed to produce the forces necessary to keep the gait deviations small (Fig 6), resulting in compensations by other muscles that exceeded the energy saved by lacking the five ankle muscles. Muscle strength here was manipulated at the whole-body or whole-limb level, so we cannot infer with confidence the effect of weakness in a specific muscle or muscle group. The literature is inconsistent on which muscles of which limbs are weak in individuals with limb loss, and most studies with strength data for multiple muscle groups suggest weakness throughout the residual limb, not just in a specific muscle or muscle group [38], but investigating the effects of individual muscles [17] is a promising direction for future work.

The main scientific contribution of the present study is that it suggests recent cross-sectional data on the metabolic cost of walking with and without limb loss in relatively young, healthy individuals [10,11] may generalize to a longitudinal (pre- vs. post-limb loss) situation. The main clinical contribution is the suggestion that minimizing strength loss, particularly in the residual limb, should be a major focus in rehabilitation after limb loss to maintain an economical walking gait with minimal gait deviations. Strength and general fitness are already emphasized in limb loss rehabilitation, but the present results suggest specific mobility goals this emphasis can achieve: walking with a relatively low metabolic cost and minimal gait deviations, even when using a passive prosthesis. Since the effect of strength loss on metabolic cost was still seen when only the residual limb had strength loss, maintaining strength of the residual limb and strength symmetry between limbs should be particularly emphasized. Strength training has been shown to reduce the metabolic cost of walking in individuals with transtibial limb loss [48]. The tested condition of no strength loss is a plausible and achievable goal of rehabilitation: physically active individuals with transtibial limb loss can often have no substantial strength asymmetries at the hip or knee, and similar levels of strength compared to non-amputees [39,46].

The main limitation of the present study is that the results were produced by human-inspired computer models, not from measurements on live humans. However, the model-based predictions of metabolic cost at different walking speeds closely matched experimental data for individuals both with and without limb loss (Fig 3), and the model’s gait mechanics and muscle activity also generally compared well with experimental data (Figs 4 and 5). These comparisons provide confidence in the model-based results. Relatedly, it could be argued that the post-limb loss simulations were further limited because they were generated by attempting to track gait mechanics data from individuals without limb loss, rather than attempting to track data from individuals with limb loss. This modeling decision was deliberate and was intended to simulate the common goal in rehabilitation and prosthesis fitting of minimizing deviations from “normal” (non-amputee) walking gait. The gait deviations in the simulations using the passive prosthesis (Fig 4) were similar to those typically seen in unilateral transtibial amputees: no major deviations in the intact limb, and greater late-swing hip flexion, less late-stance knee flexion, less late-stance ankle plantarflexion, and smaller late-stance GRF in the residual limb [49], suggesting this approach was reasonable for simulating realistic limb loss gaits even though gait data from individuals with limb loss were not involved in the simulation process.

With the exception of the ankle angle on the prosthetic/residual limb, the post-limb loss model was able to closely track the experimental gait data from individuals without limb loss in most other cases (Fig 4). While these results do not imply that tracking the non-limb loss experimental data reduced the metabolic cost of the post-limb loss model, they suggest that small gait deviations and a normal metabolic cost can be achieved concurrently when using a passive prosthesis, provided a patient has sufficient muscle strength. Relatedly, the simulations of prosthesis type and optimized ankle stiffness speak to the relative importance of modifiable factors that can contribute to the metabolic cost of walking post-limb loss. With 10% strength loss, the post-limb loss model was unable to walk with the pre-limb loss metabolic cost when using a passive prosthesis. The powered prosthesis maintained metabolic costs at or below the pre-limb loss cost for all subjects with strength losses of 10% and 20%, but not for strength loss of 30% (Fig 7). Lower metabolic costs were seen when the optimizer adjusted the prosthetic ankle stiffness on a subject-specific basis, as would be expected clinically from the process of adjusting prosthesis settings, but these costs were still above the pre-limb loss cost for all but one subject. These results suggest that maintaining muscle strength, particularly in the residual limb, may be more important than prosthesis type and settings for preventing an increase in metabolic cost following limb loss. Beyond 20–30% strength loss in the residual limb, even a powered prosthesis could not prevent a large increase in metabolic cost post-limb loss. These conclusions are limited by the fact that we only studied a simple prosthesis model, with linear ankle stiffness. Tuning a nonlinear stiffness or other prosthesis design features could potentially provide greater energy savings. For example, the toe joint was a mechanical energy sink for the both intact and residual limbs, consistent with findings on the biological feet [50,51]. Perhaps a prosthetic foot with nonlinear stiffness could reduce energy losses or increase the elastic strain energy returned by the foot during stance, to assist with ankle push-off.

The present results agree with the conclusion from the model-based study of Fey et al. [8] and the experimental study of Major et al. [4] that passive prosthesis stiffness can have a modest effect on metabolic cost (Fig 7), but add the suggestion that prosthesis stiffness is unlikely to offset increases in metabolic cost due to strength loss. The present results also agree with the model-based study of Handford and Srinivasan [9] that a powered prosthesis can in theory reduce the metabolic cost of walking below that of individuals without limb loss, and add the suggestion that this result may only be achievable with sufficient muscle strength. However, when using a passive prosthesis, the present results conflict with [9], who found that the metabolic cost of walking with a passive prosthesis was about 20% greater than their pre-limb loss model’s cost. The difference is most likely due to different cost functions in the two studies (simulations in [9] did not include a tracking term) and model formulations. The present muscle model included some features that were not present in [9] that would be expected to affect metabolic cost (e.g. activation dynamics and a force-length relationship). Nonetheless, both studies support the conclusion that a passive prosthesis cannot reduce the metabolic cost below the pre-limb loss metabolic cost.

The finding that a powered prosthesis can reduce metabolic cost below the cost of walking with a passive prosthesis agrees with one recent experimental study on a powered prosthesis [5] but disagrees with two others [6,7]. The different results between these three experimental studies could be due to differences in their device designs, or to differences in the scaling of metabolic cost (it is unclear if prosthesis mass was included in the scaling for these studies). The present study also represents a hypothetical case of ideal goal-directed motor learning (minimize gait deviations plus metabolic cost), while subjects in the experimental studies may have been unpracticed with the novel powered device, or may have been optimizing their gaits on factors other than gait deviations and metabolic cost, such as comfort or balance. Trade-offs between different criteria in multi-objective optimization of limb loss gait have recently been investigated [15], and although exploring such trade-offs was not a goal of the present study, the results may be relevant for explaining motor behavior in individual with limb loss. For any given simulation in the present work, more accurate tracking of the target gait data (i.e. smaller gait deviations) comes at the cost of greater muscle activations, which typically increases metabolic cost. With strength loss, the task of minimizing gait deviations becomes more difficult and requires greater metabolic cost (Figs 6 and 7). By extension, a potential explanation for why individuals with unilateral limb loss tend to walk asymmetrically is that an asymmetric gait is energy-optimal for their asymmetric system, or at least costs less energy than a more symmetric gait. If a clinical goal is to reduce both metabolic cost and gait asymmetry, these goals may conflict if the individual has substantial strength asymmetry.

Additional limitations of the present study include the use of a 2D model, and the modeling of a rigid residual limb-prosthesis interface. The 2D model could affect the relevance of the results if non-sagittal mechanics are primarily responsible for the energetics of gait post-limb loss. However, in high-functioning individuals with unilateral transtibial limb loss, the largest gait deviations and the majority of significant gait deviations occur in the sagittal plane [52]. We would therefore not expect fundamentally different results with a 3D model. Concerning the rigid interface assumption, soft exoskeletons store and return substantial amounts of mechanical energy in the user-device interfaces during walking [53], which ostensibly affects metabolic cost. The authors are unaware of similar analyses on transtibial prostheses, and individuals using ankle exoskeletons do not walk with the same gait mechanics as individuals using transtibial prostheses [54], so their results should be conflated with caution. Different limb-prosthesis interfaces (vacuum suspension vs. hypobaric) did not affect metabolic cost of walking in Brunelli et al. [55], but it was unclear how or if the interfaces differed in rigidity. A non-rigid prosthesis interface would likely act as a mechanical energy sink, assuming the interface is not entirely elastic, which would likely shift the data in Fig 7 upwards, but would be unlikely to re-order the data between conditions since the same sink would be present in all conditions. However, the modeling details of the residual limb-prosthesis interface are worthwhile to investigate in future work as osteointegrated prostheses with more rigid attachments become more common.

## Conclusion

The present simulation results support recent experimental data suggesting an increase in the metabolic cost of walking is not an inevitable consequence of unilateral transtibial limb loss: an increase in metabolic cost may be driven more by loss of strength in the remaining muscles rather than loss of the limb. Relatedly, while prosthesis type, design, and settings can clearly affect the metabolic cost of walking in individuals with limb loss, muscle strength can potentially have just as large of an effect, and can potentially limit the improvements in metabolic cost achievable by prosthesis adjustments.

## Supporting information

S1 File QuickTime movie comparing subject 01’s kinematics for the pre-limb loss simulation and the post-limb loss simulation with the passive prosthesis and no strength loss. (MOV)

S2 File QuickTime movie comparing subject 01’s kinematics for the pre-limb loss simulation and the post-limb loss simulation with the powered prosthesis and no strength loss. (MOV)

S3 File Appendices with model technical details and optimization details. (PDF)

## References

[1] Gailey R, Allen K, Castles J, Kucharik J, Roeder M. Review of secondary physical conditions associated with lower-limb amputation and long-term prosthesis use. J Rehabil Res Dev. 2008;45:15–30. 18566923 10.1682/jrrd.2006.11.0147

[2] Waters RL, Perry J, Antonelli D, Hislop H. Energy cost of walking of amputees: the influence of the level of amputation. J Bone Joint Surg. 1976;58A:42–46. 1249111

[3] Hafner BJ, Sanders JE, Czerniecki J, Fergason J. Energy storage and return prostheses: does patient perception correlate with biomechanical analysis? Clin Biomech. 2002;17:325–344. 10.1016/s0268-0033(02)00020-7 12084537

[4] Major MJ, Twiste M, Kenney LP, Howard D. The effects of prosthetic ankle stiffness on ankle and knee kinematics, prosthetic limb loading, and net metabolic rate of trans-tibial amputee gait. Clin Biomech. 2014;29:98–104. 10.1016/j.clinbiomech.2013.10.012 24238976

[5] Herr HM, Grabowski AM. Bionic ankle-foot prosthesis normalizes walking gait for persons with leg amputation. Proc R Soc B. 2012;279:457–464. doi: 10.1098/rspb.2011.1194 21752817 10.1098/rspb.2011.1194 PMC3234569

[6] Quesada RE, Caputo JM, Collins SH. Increasing ankle push-off work with a powered prosthesis does not necessarily reduce metabolic rate for transtibial amputees. J Biomech. 2016;49:3452–3459. doi: 10.1016/j.jbiomech.2016.09.015 27702444 10.1016/j.jbiomech.2016.09.015

[7] Gardinier ES, Kelly BM, Wensman J, Gates DH. A controlled clinical trial of a clinically-tuned powered ankle prosthesis for people with transtibial amputation. Clin Rehabil. 2017 7 27 pii: 269215517723054. doi: 10.1177/0269215517723054 28750586 10.1177/0269215517723054

[8] Fey NP, Klute GK, Neptune RR. Optimization of prosthetic foot stiffness to reduce metabolic cost and intact knee loading during transtibial amputee walking: a theoretical study. J Biomech Engin. 2012;134:111005. 10.1115/1.4007824 PMC3707817 23387787

[9] Handford ML, Srinivasan M. Robotic lower limb prosthesis design through simultaneous computer optimizations of human and prosthesis costs. Sci Rep. 2016;6:19983 doi: 10.1038/srep19983 26857747 10.1038/srep19983 PMC4746571

[10] Russell Esposito E, Rodriguez KM, Ràbago CA, Wilken JM. Does unilateral transtibial amputation lead to greater metabolic demand during walking? J Rehabil Res Dev. 2014;51:1287–1296. doi: 10.1682/JRRD.2014.06.0141 25671680 10.1682/JRRD.2014.06.0141

[11] Jarvis HL, Bennett AN, Twiste M, Phillip RD, Etherington J, Baker R. Temporal spatial and metabolic measures of walking in highly functional individual with lower limb amputations. Arch Phys Med Rehabil. 2017;98:1389–1399. doi: 10.1016/j.apmr.2016.09.134 27865845 10.1016/j.apmr.2016.09.134

[12] Ziegler-Graham K, MacKenzie EJ, Ephraim PL, Travison TG, Brookmeyer R. Estimating the prevalence of limb loss in the United States: 2005 to 2050. Arch Phys Med Rehabil. 2008;89:422–429. doi: 10.1016/j.apmr.2007.11.005 18295618 10.1016/j.apmr.2007.11.005

[13] Umberger BR, Rubenson J. Understanding muscle energetics in locomotion: new modeling and experimental approaches. Exerc Sport Sci Rev. 2011;39:59–67. doi: 10.1097/JES.0b013e31820d7bc5 21206279 10.1097/JES.0b013e31820d7bc5

[14] Umberger BR, Gerritsen KGM, Martin PE. Muscle fiber type effects on energetically optimal cadences in cycling. J Biomech. 2006;39:1472–1479. doi: 10.1016/j.jbiomech.2005.03.025 15923008 10.1016/j.jbiomech.2005.03.025

[15] Koelewijn AD, van den Bogert AJ. Joint contact forces can be reduced by improving joint moment symmetry in transtibial amputee gait simulations. Gait Posture. 2016;49:219–225. doi: 10.1016/j.gaitpost.2016.07.007 27459416 10.1016/j.gaitpost.2016.07.007

[16] Zmitrewicz RJ, Neptune RR, Sasaki K. Mechanical energetic contributions from individual muscles and elastic prosthetic feet during symmetric unilateral transtibial amputee walking: a theoretical study. J Biomech. 2007;40:1824–1831. doi: 10.1016/j.jbiomech.2006.07.009 17045595 10.1016/j.jbiomech.2006.07.009

[17] Silverman AK, Neptune RR. Muscle and prosthesis contributions to amputee walking mechanics: a modeling study. J Biomech. 2012;45:2271–2278. doi: 10.1016/j.jbiomech.2012.06.008 22840757 10.1016/j.jbiomech.2012.06.008

[18] Silverman AK, Neptune RR. Three-dimensional knee joint contact forces during walking in unilateral transtibial amputees. J Biomech. 2014;47:2556–2562. doi: 10.1016/j.jbiomech.2014.06.006 24972921 10.1016/j.jbiomech.2014.06.006

[19] Pickle NT, Grabowski AM, Jeffers JR, Silverman AK. The functional roles of muscles, passive prostheses and powered prostheses during sloped walking in people with a transtibial amputation. J Biomech Engin. 2017;139:111005. 10.1115/1.4037938 PMC5676650 28975280

[20] Highsmith MJ, Andrews CR, Millman C, Fuller A, Kahle JT, Klenow TD, et al Gait training interventions for lower extremity amputees: a systematic literature review. Technol Innov. 2016;18:99–113. doi: 10.21300/18.2-3.2016.99 28066520 10.21300/18.2-3.2016.99 PMC5218520

[21] Jonkergouw N, Prins MR, Buis AW, Wurff PV. The effect of alignment changes on unilateral transtibial amputee’s gait: a systematic review. PLoS ONE. 2016;11:e0167466 doi: 10.1371/journal.pone.0167466 27923050 10.1371/journal.pone.0167466 PMC5140067

[22] Miller RH, Hamill J. Optimal footfall patterns for cost minimization in running. J Biomech. 2015;48:2858–2864. doi: 10.1016/j.jbiomech.2015.04.019 25952545 10.1016/j.jbiomech.2015.04.019

[23] Anderson DE, Madigan ML, Nussbaum MA. Maximum voluntary joint torque as a function of joint angle and angular velocity: model development and application to the lower limb. J Biomech. 2007;40:3105–3113. doi: 10.1016/j.jbiomech.2007.03.022 17485097 10.1016/j.jbiomech.2007.03.022 PMC6820133

[24] Minetti AE, Alexander RM. A theory of metabolic costs for bipedal gaits. J Theor Biol. 1997;186:467–476. doi: 10.1006/jtbi.1997.0407 9278722 10.1006/jtbi.1997.0407

[25] Riener R, Edrich T. Identification of passive elastic joint moments in the lower extremities. J Biomech. 1999;32:539–544. 10327008 10.1016/s0021-9290(99)00009-3

[26] Lehmann JF, Price R, Boswell-Bessette S, Dralle A, Questad K, deLateur BJ. Comprehensive analysis of energy storing prosthetic feet: Flex Foot and Seattle Foot versus standard SACH foot. Arch Phys Med Rehabil. 1993;74:1225–1231. 8239969

[27] Smith JD, Martin PE. Effects of prosthetic mass and mass distribution on metabolic costs and walking symmetry. J Appl Biomech. 2013;29:317–328. 22977207 10.1123/jab.29.3.317

[28] Scovil CY, Ronsky JL. Sensitivity of a Hill-based muscle model to perturbations in model parameters. J Biomech. 2006;39:2055–2063. doi: 10.1016/j.jbiomech.2005.06.005 16084520 10.1016/j.jbiomech.2005.06.005

[29] Ackland DC, Lin YC, Pandy MG. Sensitivity of model predictions of muscle function to changes in moment arms and muscle-tendon properties: a Monte-Carlo analysis. J Biomech. 2012;45:1463–1471. doi: 10.1016/j.jbiomech.2012.02.023 22507351 10.1016/j.jbiomech.2012.02.023

[30] Hasson CJ, Caldwell GE. Effects of age on mechanical properties of dorsiflexor and plantarflexor muscles. Ann Biomed Engin. 2012;40:1088–1101. 10.1007/s10439-011-0481-4 22187136

[31] Neptune RR, Hull ML. Evaluation of performance criteria for simulation of submaximal steady-state cycling using a forward dynamic model. J Biomech Engin. 1998;120:334–341. 10.1115/1.2797999 10412400

[32] Van den Bogert AJ, Blana D, Heinrich D. Implicit methods for efficient musculoskeletal simulation and optimal control. Proceedia IUTAM. 2011;2:297–316. 10.1016/j.piutam.2011.04.027 PMC3217276 22102983

[33] Miller RH, Edwards WB, Brandon SCE, Morton AM, Deluzio KJ. Why don’t most runners get knee osteoarthritis? A case for per-unit-distance loads. Med Sci Sports Exerc. 2014;46:572–579. doi: 10.1249/MSS.0000000000000135 24042311 10.1249/MSS.0000000000000135

[34] Fraisse N, Martinet N, Kpadonou TJ, Paysant J, Blum A, André JM. Muscles of the below-knee amputee. Ann Readapt Med Phys. 2008;51:218–227. doi: 10.1016/j.annrmp.2008.01.012 18358554 10.1016/j.annrmp.2008.01.012

[35] Lanza IR, Towse TF, Caldwell GE, Wigmore DM, Kent-Braun JA. Effects of age on human muscle torque, velocity, and power in two muscle groups. J Appl Physiol. 2003;95:2361–2369. doi: 10.1152/japplphysiol.00724.2002 12923120 10.1152/japplphysiol.00724.2002

[36] South BJ, Fey NP, Bosker G, Neptune RR. Manufacture of energy storage and return prosthetic feet using selective laser sintering. J Biomech Engin. 2010;132:015001. 10.1115/1.4000166 20524754

[37] Umberger BR, Gerritsen KGM, Martin PE. A model of human muscle energy expenditure. Comput Methods Biomech Biomed Engin. 2003;6:99–111. doi: 10.1080/1025584031000091678 12745424 10.1080/1025584031000091678

[38] Moirenfeld I, Ayalon M, Ben-Sira D, Isakov E. Isokinetic strength and endurance of the knee extensors and flexors in trans-tibial amputees. Prosthet Orthot Int. 2000;24:221–225. doi: 10.1080/03093640008726551 11195357 10.1080/03093640008726551

[39] Nolan L. Lower limb strength in sports-active transtibial amputees. Prosthet Orthot Int. 2009;33:230–241. doi: 10.1080/03093640903082118 19658013 10.1080/03093640903082118

[40] Cohen J. Statistical power analysis for the behavioral sciences. 2nd ed. London: Routledge, 1988.

[41] Rouder JN, Speckman PL, Sun D, Morey RD. Bayesian t tests for accepting and rejecting the null hypothesis. Psychon Bull Rev. 2009;16:225–237. doi: 10.3758/PBR.16.2.225 19293088 10.3758/PBR.16.2.225

[42] Jeffreys H. The theory of probability. 3rd ed. Oxford: Clarendon Press, 1961.

[43] Sutherland DH. The evolution of clinical gait analysis part I: kinesiological EMG. Gait Posture. 2001;14:61–70. 11378426 10.1016/s0966-6362(01)00100-x

[44] Cromwell RL, Aadland-Monahan TK, Nelson AT, Stern-Sylvestre SM, Seder B. Sagittal plane analysis of head, neck, and trunk kinematics and electromyographic activity during locomotion. J Orthop Sport Phys Ther. 2001;31:255–262. 10.2519/jospt.2001.31.5.255 11352192

[45] Rubenson J, Heliams DB, Maloney SK, Withers PC, Lloyd DG, Fourner PA. Reappraisal of the comparative cost of human locomotion using gait-specific allometric analyses. J Exp Biol. 2007;210:3513–3524. doi: 10.1242/jeb.000992 17921153 10.1242/jeb.000992

[46] Bäcklund L, Lemperg R, Ottosson LG. Leg muscle strength in transtibial amputees. Acta Orthop Scand. 1968;39:107–116. 5730103 10.3109/17453676808989445

[47] DeVita P, Helseth J, Hortobagyi T. Muscles do more positive than negative work in human locomotion. J Exp Biol. 2007;210:3361–3373. doi: 10.1242/jeb.003970 17872990 10.1242/jeb.003970 PMC2577758

[48] Nolan L. A training programme to improve hip strength in persons with lower limb amputation. J Rehabil Med. 2012;44:241–248. doi: 10.2340/16501977-0921 22367416 10.2340/16501977-0921

[49] Sanderson DJ, Martin PE. Lower extremity kinematic and kinetic adaptations in unilateral transtibial amputees during walking. Gait Posture. 1997;6:126–136.

[50] Zelik KE, Takahashi KZ, Sawicki GS. Six degree of freedom analysis of hip, knee, ankle and foot provides updated understanding of biomechanical work during human walking. J Exp Biol. 2015;218:876–886. doi: 10.1242/jeb.115451 25788726 10.1242/jeb.115451

[51] Honert EC, Zelik KE. Inferring muscle-tendon unit power from ankle joint power during the push-off phase of human walking: insights from a multiarticular EMG-driven model. PLoS ONE. 2016;11:e0163169 doi: 10.1371/journal.pone.0163169 27764110 10.1371/journal.pone.0163169 PMC5072599

[52] Rábago CA, Wilken JM. The prevalence of gait deviations in individuals with transtibial amputation. Mil Med. 2016;181:30–37. doi: 10.7205/MILMED-D-15-00505 27849459 10.7205/MILMED-D-15-00505

[53] Yandell MB, Quinlivan BT, Popov D, Walsh C, Zelik KE. Physical interface dynamics alter how robotic exosuits augment human movement: implications for optimizing wearable assistive devices. J Neuroeng Rehabil. 2017;14:40 doi: 10.1186/s12984-017-0247-9 28521803 10.1186/s12984-017-0247-9 PMC5437613

[54] Russell Esposito E, Stinner DJ, Fergason JR, Wilken JM. Gait biomechanics following lower extremity trauma: amputation vs. reconstruction. Gait Posture. 2017;54:167–173. doi: 10.1016/j.gaitpost.2017.02.016 28314214 10.1016/j.gaitpost.2017.02.016

[55] Brunelli S, Delussu AS, Paradisi F, Pellegrinni R, Traballesi M. A comparison between the suction suspension system and the hypobaric Iceross Seal-In® X5 in transtibial amputees. Prosthet Orthot Int. 2013;37:436–444. doi: 10.1177/0309364613476531 23436696 10.1177/0309364613476531

[56] Delp SL, Anderson FC, Arnold AS, Loan P, Habib A, John CT, Guendelman E, Thelen DG. OpenSim: open-source software to create and analyze dynamic simulations of movement. IEEE Trans Biomed Engin. 2007;54:1940–1950. 10.1109/TBME.2007.901024 18018689

