# Supplementary material for: Maintenance of muscle strength retains a normal metabolic cost in simulated walking after transtibial limb loss
Source: PLoS One. 2018 Jan 12;13(1):e0191310. doi: 10.1371/journal.pone.0191310 (PMC5766241; doi:10.1371/journal.pone.0191310)
Supplement: S3 File — (PDF) [file pone.0191310.s003.pdf]

Appendix A: model technical details

The model (Fig 1) was a mechanical linkage with 12 generalized coordinates: the position and orientation of the pelvis, and the angles of the lumbar, hip, knee, ankle, and toe joints. All segments were one-dimensional rigid links with the exception of the foot, which was two-dimensional. All joints were pin joints. The model had a total body mass of 73.0 kg and stature 1.83 m. Segment inertial properties were defined for a healthy young adult male according to de Leva (1996). The model's state equations were defined in MG Kane software (Motion Genesis, Sunnyvale, CA, USA) and exported to a custom Matlab code. The equations of motion were:

$$\mathbf{M}(\mathbf{q})\ddot{\mathbf{q}} + \mathbf{B}(\mathbf{q},\dot{\mathbf{q}}) + \frac{\partial \mathbf{L}(\mathbf{q})}{\partial \mathbf{q}}\mathbf{F} = 0 \tag{1}$$

where $\mathbf{M}(\mathbf{q})$ is the system mass matrix, $\mathbf{q}$ is the generalized coordinates, $\mathbf{B}(\mathbf{q},\dot{\mathbf{q}})$ is the generalized forces due to gravity, centrifugal and Coriolis effects, foot-ground contact, and passive joint stiffness, $\mathbf{L}(\mathbf{q})$ is the muscle origin-to-insertion lengths, and $\mathbf{F}$ is the muscle forces. Muscle lengths were defined as linear functions of the joint angles using data from Menegaldo et al. (2004), and muscle moment arms were then defined using the virtual work principle (An et al., 1984).

Foot-ground contact

Deformable foot-ground contact was modeled by defining 11 discrete contact elements on the plantar surface of each foot, spaced equally between the heel and toe-tip. Each element produced a vertical force F_y as a cubic spring with multiplicative damping and a horizontal force F_x as a Coulomb friction approximation:

$$F_y = A_y d_y^2 (1 - B_y \dot{y}) \tag{2}$$

$$d_y = \frac{1}{2}\left(\sqrt{y^2 + y_0^2} - y\right) \tag{3}$$

$$F_x = -\mu F_y \tanh(\dot{x}/\gamma) \tag{4}$$

where x and y are the horizontal and vertical coordinates of the contact element. The contact model parameters $A_y = 2.0 \cdot 10^6$ N/m^2, $B_y = 1.0$ Ns/m, $y_0 = 0.001$, $\mu = 1.0$, and $\gamma = 0.05$ m/s were set to produce realistic force-deformation loops and shear forces for shod human feet during walking and running (Miller & Hamill, 2015; Miller et al., 2015).

Passive joint moments

The hip, knee, and ankle joints were softly constrained to physiological ranges of motion by passive torques τ that were exponential functions of the joint angles:

$$\tau = \tau_0 - e^{k_1 + k_2\theta_H + k_3\theta_K + k_4\theta_A} + e^{k_5 + k_6\theta_H + k_7\theta_K + k_8\theta_A} + c \cdot e^{2.22 + 8.5944\theta_K} \tag{5}$$

where θ_H, θ_K, and θ_A are the hip, knee, and ankle angles and the other variables in Eq. 5 are joint-specific constants based on dynamometry measurements from healthy young adult males (Riener & Edrich, 1999). The lumbar and toe joints had linear passive torque-angle relationships, with respective stiffnesses of 150 Nm/rad and 5 Nm/rad based on our previous work (Miller et al., 2015).

Muscle forces

Each muscle model consisted of a contractile component (CC) in series with an elastic component (SEC) (Fig. A1). The CC received an input time-varying excitation signal $u(t)$ and in

response produced force in accordance with its activation dynamics, force-length, and force-velocity relationships. This force was expressed on the SEC, which responded elastically in accordance with its force-extension relationship. The CC activation dynamics were (He et al., 1991):

$$\dot{\alpha} - (u - \alpha)(c_1 u + c_2) = 0 \tag{6}$$

$$c_1 = 1/(0.080 - 0.050FT) - 1/(0.095 - 0.060FT) \tag{7}$$

$$c_2 = 1/(0.095 - 0.060FT) \tag{8}$$

where α is the muscle activation level and FT is the fraction of fast-twitch muscle fibers (Umberger et al., 2003). The muscle contractile dynamics were:

$$(F_o \cdot \alpha \cdot FL \cdot FV) - F_{SEC}(\lambda, \mathbf{q}) = 0 \tag{9}$$

where F_o is the maximum isometric force, FL and FV are the force-length and force-velocity factors, ϕ is the CC pennation angle, λ is the CC length, and $F_{SEC}(\lambda, \mathbf{q})$ is the SEC force. The CC force-length relationship was (Winters and Stark, 1985):

$$FL = \exp\left(-\frac{1}{W}\left(\frac{\lambda}{L_\alpha} - 1\right)^2\right) \tag{10}$$

$$L_\alpha = L_o(\gamma(1 - \alpha) + 1) \tag{11}$$

where W is a muscle-specific constant that affects the width of the curve, L_α is the optimal CC length at an arbitrary activation level, L_o is the optimal CC length at maximum activation, and $\gamma = 0.15$ shifts the optimal CC length to longer lengths at lower activation levels (Lloyd & Besier, 2003). The CC force-velocity relationship was (Hatze, 1977):

$$FV = \frac{C_{ecc}}{2}\left(1 + \tanh\left(a\left(\frac{\dot{\lambda}}{v_{max}L_o} - b\right)\right)\right) \tag{12}$$

$$a = 2.3434 - 0.9911 \ln FT \tag{13}$$

$$b = -\frac{\tanh(2/C_{ecc} - 1)}{a} \tag{14}$$

where $C_{ecc} = 1.5$ is the eccentric plateau, $v_{max} = 12$ is the maximum shortening velocity, and FT is the fraction of fast-twitch fibers. Equation 13 approximates the sensitivity of force-velocity curvature to fiber type from Umberger et al. (2003). Equation 14 ensures that $FV = 1$ when $\dot{\lambda} = 0$. The SEC force was (Caldwell, 1995):

$$F_{SEC} = F_o C_{SEC}\left(\exp\left(K_{SEC}\left(\frac{L(\mathbf{q}) - \lambda}{L_u} - 1\right)\right) - 1\right) \tag{15}$$

where $C_{SEC} = 0.0258$ and $K_{SEC} = 92.08$ approximate the SEC force-extension relationship from Bahler (1967), $L(\mathbf{q})$ is the muscle origin-to-insertion length, and L_u is the unloaded SEC length.

The muscle's rate of metabolic energy expenditure was based on the empirical model of Minetti and Alexander (1997):

$$\dot{E} = m \cdot \alpha^n \cdot \left(\frac{0.054 - 0.506\left(\frac{\dot{\lambda}}{v_{max}L_o}\right) + 2.46\left(\frac{\dot{\lambda}}{v_{max}L_o}\right)^2}{1 + 1.13\left(\frac{\dot{\lambda}}{v_{max}L_o}\right) + 12.8\left(\frac{\dot{\lambda}}{v_{max}L_o}\right)^2 + 1.64\left(\frac{\dot{\lambda}}{v_{max}L_o}\right)^3}\right) \tag{16}$$

where m is the muscle's mass. A value of $n = 1.1$ was found to produce realistic metabolic costs with realistic timing and distribution of muscle activity, and is motivated by the nonlinear scaling of the heat rate in active skeletal muscle with activation level (Umberger et al., 2003).

Appendix B: optimization details

The optimal control problems were solved using an implicit direct collocation method (van den Bogert et al., 2011; Miller & Hamill, 2015). The model's state variables were stacked into the state vector $\mathbf{x}$, and its state equations (Eqs. 1, 6, and 9) were stacked into the dynamics vector $\mathbf{f}$:

$$\mathbf{f}(\mathbf{x}, \dot{\mathbf{x}}, \mathbf{u}) = 0 \quad (17)$$

$$\mathbf{x} = [\mathbf{q}; \dot{\mathbf{q}}; \boldsymbol{\alpha}; \boldsymbol{\lambda}] \quad (18)$$

where $\mathbf{u}$ is the vector of all muscle excitations, $\boldsymbol{\alpha}$ is the vector of all muscle activations, and $\boldsymbol{\lambda}$ is the vector of all CC lengths. The constraints on the solution domain were the system dynamics (Eq. 17), periodicity in the states with average horizontal speed v (Eq. 19), periodicity in the controls (Eq. 20), and physiological bounds on the controls (Eq. 21):

$$\mathbf{x}(T) - \mathbf{x}(0) - \hat{\mathbf{x}}_1 vT = 0 \quad (19)$$

$$\mathbf{u}(T) - \mathbf{u}(0) = 0 \quad (20)$$

$$0 \leq \mathbf{u}(t) \leq 1 \quad (21)$$

where $\hat{\mathbf{x}}_1$ is a unit vector in the direction of horizontal pelvis translation.

The states, controls, cost function, and constraints were discretized on a temporal grid of 101 nodes spaced equally over the interval $t \in [0, T]$ using the Backward Euler method. Discretization converted the optimal control problem to a nonlinear programming problem, which was solved numerically using IPOPT (Wächter & Biegler, 2006). When using the pre-limb loss model, the optimization was first performed on a 21-node grid starting from an initial guess of upright standing. This solution was then interpolated as the initial guess onto a 101-node grid. Finer grids up to 301 nodes affected the difference in metabolic costs between the pre- and post-limb loss models by under 1%. When using the post-limb loss model, the pre-limb loss model's 101-node solution was used as the initial guess.

Optimizations were performed on an "Early 2015" model MacBook Pro with a 3.1-GHz Intel Core i7 CPU and 16 GB of RAM (Apple Inc., Cupertino, CA, USA). Optimizations on the 101-node grid typically required about 30 minutes for convergence on this machine. The convergence settings were 10^{-2} for the scaled NLP error tolerance and 10^{-4} for the constraint tolerance, which corresponded to acceptable violations of the implicit equations of motion (Eq. 1) of 0.01 Nm and violations of the muscle contractile dynamics (Eq. 9) of 0.01% of F_o.

References

An KN, Takahashi K, Harrigan TP, Chao EY. Determination of muscle orientations and moment arms. *J Biomech Engin*. 1984;**106**:280-282.

Bahler AH. Series elastic component of mammalian skeletal muscle. *Am J Physiol*. 1967;**213**:1560-1564.

Caldwell GE. Tendon elasticity and relative length: effects on the Hill two-component muscle model. *J Appl Biomech*. 1995;**11**:1-24.

De Leva P. Adjustments to Zatsiorsky-Seluyanov's segment inertia parameters. *J Biomech*. 1996;**29**:1223-1230.

Hatze H. A myocybernetic control model of skeletal muscle. *Biol Cybern*. 1977;**25**:103-119.

He J, Levine WS, Loeb GE. Feedback gains for correcting small perturbations to standing posture. *IEEE Trans Autom Control*. 1991;**36**:322-332.

Lloyd DG, Besier TF. An EMG-driven musculoskeletal model to estimate muscle forces and knee joint moments in vivo. *J Biomech*. 2003;**36**:765-776.

Menegaldo LL, de Toledo Fleury A, Webrer HI. Moment arms and musculotendon lengths estimation for a three-dimensional lower-limb model. *J Biomech*. 2004;**37**:1447-1453.

Miller RH, Esterson AY, Shim JK. Joint contact forces when minimizing the external knee adduction moment by gait modification: a computer simulation study. *Knee*. 2015;**22**:481-489.

Miller RH, Hamill J. Optimal footfall patterns for cost minimization in running. *J Biomech*. 2015;**48**:2858-2864.

Minetti AE, Alexander RM. A theory of metabolic costs for bipedal gaits. *J Theor Biol*. 1997;**186**:467-476.

Riener R, Edrich T. Identification of passive elastic joint moments in the lower extremities. *J Biomech*. 1999;**32**:539-544.

Umberger BR, Gerritsen KGM, Martin PE. A model of human muscle energy expenditure. *Comput Methods Biomech Biomed Engin*. 2003;**6**:99-111.

Wächter A, Biegler LT. On the implementation of an interior-point line-search algorithm for large-scale nonlinear programming. *Math Prog A*. 2006;**106**:25-57.

Winters JM, Stark L. Analysis of fundamental human movement patterns through the use of in-depth antagonistic muscle models. *IEEE Trans Biomed Engin*. 1985;**32**:826-839.

Van den Bogert AJ, Blana D, Heinrich D. Implicit methods for efficient musculoskeletal simulation and optimal control. *Proceedia IUTAM*. 2011;**2**:297-316.
